# Transforming Emergency Care Through Telemedicine: A Narrative Review

**DOI:** 10.7759/cureus.98481

**Published:** 2025-12-04

**Authors:** Courage O Idahor, Olamide Ogunfuwa, Ndidiamaka Ogbonna, Omo A Ogbeide, Opeyemi Abe, Ojeyemi Oore-ofe

**Affiliations:** 1 Emergency Medicine, Nottingham University Hospitals NHS Trust, Nottingham, GBR; 2 Emergency Medicine, Sandwell and West Birmingham Hospitals NHS Trust, Birmingham, GBR; 3 Family Medicine, Edo State University Uzairue, Uzairue, NGA; 4 Hospital-Based Medicine, Chaucer Hospital, Canterbury, GBR; 5 Psychiatry, Priory Hospital Cheadle Royal, Cheadle, GBR; 6 General Practice, Lagoon Hospital, Lagos, NGA

**Keywords:** covid-19, emergency department, emergency medicine and trauma, telemedicine (tm), triage

## Abstract

Telemedicine has fleetly evolved from a niche result to a central pillar in modern emergency and critical care systems. This narrative review delves into the multifaceted role of telemedicine in emergency settings, tracing its historical development, present applications, and future possibilities. It examines how telemedicine islands geographical and infrastructural gaps, particularly in underserved communities, by enabling timely access to specialist care similar to telestroke services and remote ferocious care. Substantiation highlights advancements in clinical outcomes, functional effectiveness, and patient satisfaction, with global case studies demonstrating successful perpetration across both high- and low-resource settings.

Despite these advances, challenges persist. Technological restrictions, regulatory barriers, digital knowledge gaps, and unlikeness in assent continue to hamper wide relinquishment. This review discusses these obstacles and underscores the significance of strategic investment, cross-sector collaboration, and policy reform. Arising inventions, including artificial intelligence, wearable devices, and scalable telehealth platforms, signal promising directions for perfecting reach and adaptability in emergency systems.

Additionally, this paper identifies crucial areas for unborn research, including long-term outgrowth assessment and telemedicine's part in disaster and pandemic response. By synthesizing current substantiation and practical perceptivity, this review aims to inform clinicians, health system leaders, and policymakers about the transformative eventuality and ongoing challenges of telemedicine in emergency care. Eventually, it calls for sustained invention, equity-concentrated perpetration, and cooperative sweats to completely realize telemedicine's pledge in erecting a more accessible, responsive, and flexible emergency care geography.

## Introduction and background

Telehealth has surfaced as a prominent topic of discussion in recent years, particularly in the environment of the COVID-19 pandemic, which heightened the need for and reliance on remote healthcare services. Accordingly, telehealth and telemedicine have become focal points for healthcare associations and providers [1]. The United States Department of Health and Human Services (HHS) defines telehealth as a modality that enables physicians to deliver care without taking in-person office visits, primarily through internet-enabled devices such as computers, tablets, or smartphones [1,2]. 

At first glance, the high-perceptivity and time-sensitive nature of emergency medicine may feel inharmonious with telehealth, as emergency medicine traditionally relies on immediate, hands-on intervention. Nevertheless, the specialty has progressively integrated telehealth technologies to enhance patient care and optimize workflows [1]. 

Emergency departments (EDs) are critical factors of contemporary healthcare systems, serving as essential points of care for acute itinerant and inpatient services [3-5]. Conditions managed within the ED account for approximately 50.7% of overall mortality and 41.5% of the global disease burden [3]. ED overcrowding is a persistent challenge that has drawn considerable attention from non-supervisory bodies and the public worldwide [3,6]. An additional burden is posed by the affluence of non-urgent and less-critical patients, frequently driven by limited patient education or confined access to alternative healthcare services [3,7]. These patients are constantly dispersed across different sections of the hospital and, when directed to EDs, contribute to overcrowding. This overcrowding can stymie the care of critically ill patients requiring resuscitation or critical intervention [3,8]. Furthermore, overcrowding may lead to lowered patient satisfaction and increased vulnerability to adverse events [3,8].

In recent years, the role of telemedicine in mitigating ED overcrowding has gained significant scholarly and clinical attention as healthcare delivery models continue to evolve [3,9]. Telemedicine has been honored as a feasible volition to traditional in-person care, with the eventuality to enhance access, ameliorate effectiveness, and reduce costs [3,10]. Advances in technology, declining equipment costs, and rising patient prospects have further accelerated the relinquishment of telemedicine across healthcare settings [5,11]. 

Telemedicine is widely applied in emergency care via means of information technologies, which has immensely improved the provision of healthcare services [5,12]. Studies have stressed its eventuality for delivering specialized services to rural areas [5,13-16], easing ED overcrowding [5,17-20] supporting paramedics, enhancing crisis management, and reducing the time between accidents and hospital admission [5,21-23]. 

The HHS identifies five crucial telehealth applications relevant to emergency medicine [1,2]: (1) tele-triage, leveraging telehealth to estimate the severity of a patient's condition and ascertain the suitable care and offers necessary; (2) tele-emergency care, connecting clinicians at centralized EDs with those at lower or rural hospitals through video conferencing or other telehealth technologies; (3) virtual rounds, allowing the remote monitoring of ED patients, thereby reducing the need for on-site healthcare providers; (4) electronic consults (e-consults), enabling healthcare providers to seek specialty consultations or management advice remotely; and (5) telehealth for follow-up care, using telehealth platforms to follow through with patients who were either triaged but not admitted or discharged from the ED.

Several studies have demonstrated that telemedicine relinquishment is associated with a reduction in non-emergent ED visits [3,24]. Virtual consultations, which allow patients to seek medical advice from the comfort of their homes, are particularly appealing for minor ailments and routine follow-ups [3,25]. Telemedicine has also been shown to be largely effective in managing chronic conditions by easing non-stop monitoring and timely interventions, thereby reducing the need for in-person visits [3,26]. However, significant barriers remain, including differences in access to technology, variations in digital literacy, and concerns over the quality of virtual care [3,27]. Despite its felicity for numerous non-urgent cases, telemedicine cannot replace in-person evaluations in all circumstances, particularly during emergencies or outbreaks such as COVID-19 [3,28]. 

Achieving a balance between the convenience offered by telemedicine and the essential need for in-person care is vital for enhancing healthcare delivery and securing patient safety. Furthermore, telemedicine's influence lengthens beyond individual clinical hassles to encompass comprehensive systemic considerations. To fully integrate telehealth into current healthcare models, policymakers and healthcare administrators must tackle challenges related to regulation, reimbursement, and infrastructure [29]. Effectively reducing non-urgent visits to EDs through telemedicine will require a holistic strategy that incorporates individual adoption, system-wide integration, and policy-position support [3,30].

## Review

Telemedicine in emergency medicine: background and context

In recent decades, telemedicine has grown from a futuristic concept into a critical component of modern healthcare systems. At its core, telemedicine refers to the use of telecommunications technology to deliver medical care over distances. Its application in emergency medicine has become especially valuable in improving access to timely and specialized care, particularly in underserved or geographically isolated settings. As healthcare systems worldwide face increasing demands, from aging populations to workforce shortages and public health crises, telemedicine offers practical solutions to improve outcomes, enhance efficiency, and bridge service gaps. Emergency medicine, with its time-sensitive nature, stands to benefit immensely from such innovation.

Historical Development

The roots of telemedicine in emergency care stretch back to the early 20th century. One of the earliest notable contributions was Willem Einthoven's transmission of electrocardiograms via telephone lines in 1906, establishing the first remote diagnostic exchange [31]. However, it wasn't until the 1960s that telemedicine began to take practical shape in emergency care. In Miami, emergency physicians collaborated with fire departments to transmit cardiac rhythms by radio from the field to the hospital, enabling physicians to guide early interventions [32]. This early system demonstrated how distance could be overcome to deliver critical care decisions in real time.

Another landmark development came in the 1970s with the Space Technology Applied to Rural Papago Advanced Health Care (STARPAHC) project, a collaboration between the National Aeronautics and Space Administration (NASA) and the Indian Health Service. This project used satellite communications to provide healthcare services to the Tohono O'odham Nation in Arizona and astronauts in space, exemplifying telemedicine's dual utility in both terrestrial and space-based emergencies [33]. These foundational efforts underscored the potential of technology to support urgent medical decision-making even in remote or extreme environments.

Through the decades, several key moments have accelerated the adoption of telemedicine in emergency contexts. The 1988 Armenian earthquake prompted the creation of the "Spacebridge to Armenia", where US and Soviet physicians jointly used satellite links to coordinate disaster relief and medical consultation [34]. In the 1990s, telestroke programs emerged as game changers. Neurologists began remotely guiding emergency room teams in the rapid assessment and management of acute strokes, resulting in faster thrombolysis and improved patient outcomes [35]. Most recently, the COVID-19 pandemic placed telemedicine in the spotlight. Hospitals worldwide implemented tele-triage systems to reduce crowding, safeguard staff, and deliver timely care while minimizing the risk of viral transmission [36].

Integration Into Emergency Systems

Telemedicine's integration into emergency systems has been two-pronged: strengthening prehospital care and augmenting in-hospital emergency responses. On the prehospital front, emergency medical services (EMS) now routinely use telemedicine to transmit critical patient data such as electrocardiograms and vital signs en route to EDs. This early notification enables hospital teams to mobilize resources before the patient's arrival, thereby expediting care [37]. Additionally, telemedicine has proven especially effective in mass casualty incidents or natural disasters. Programs like the European Space Agency's Disaster Emergency Logistic Telemedicine Advanced Satellite System (DELTASS) have used satellite telecommunication to coordinate response efforts across multiple first responder agencies [38].

Inside EDs, telemedicine has transformed care delivery through virtual triage and remote specialist consultations. With growing patient volumes and limited resources, many hospitals now use virtual triage systems to assess and prioritize incoming cases based on acuity, reducing bottlenecks and improving throughput [39]. Furthermore, teleconsultations have enabled real-time access to specialists such as neurologists, trauma surgeons, and toxicologists, especially in smaller or rural facilities where such expertise may not be physically available [40]. These innovations have led to faster decision-making, reduced transfer rates, and ultimately better patient outcomes.

Beyond clinical care, telemedicine has also found a role in the education and supervision of emergency medicine residents, especially during the COVID-19 pandemic when physical restrictions limited traditional bedside teaching [41]. This added dimension highlights telemedicine's expanding influence within the broader emergency medicine ecosystem.

Policy and Frameworks

Despite its growing utility, telemedicine in emergency care operates within a complex regulatory environment. In the United States, issues surrounding licensure, reimbursement, and patient confidentiality have historically impeded rapid expansion. To address this, the American College of Emergency Physicians (ACEP) has developed policy statements advocating for the ethical and standardized use of telehealth, including the protection of patient data and the equitable distribution of services [42]. The COVID-19 pandemic led to temporary policy shifts such as expanded Medicare reimbursement and relaxed interstate licensure rules, which have fueled ongoing debates on making these reforms permanent [43].

Globally, the adoption and regulation of telemedicine vary based on local needs and infrastructure. Sweden, for instance, has integrated telemedicine deeply into its public healthcare system, guided by long-standing data protection laws and a well-developed digital health infrastructure. Meanwhile, Singapore has adopted a targeted regulatory approach, combining ethical guidelines with legal frameworks that address clinical quality, data security, and professional accreditation. The city-state is poised to implement a comprehensive Healthcare Services Act that will govern telemedicine more formally [44]. These international perspectives emphasize the importance of adaptable and context-specific regulatory frameworks in successfully integrating telemedicine into emergency care.

Opportunities for telemedicine in emergency care

The growth of telemedicine has been driven by rising global health demands alongside market influences. In emergency and critical care, major challenges exist in accessing timely care, specialized expertise, and advanced technology. Telemedicine helps to overcome these gaps on both a global and regional scale, providing the highest level of expertise and care to the most remote settings. Telehealth has served to strengthen patient care, surgical education, and inter-institutional collaboration through the use of modern technology. 

Expanding Access to Emergency Services

Notably, the burden of emergency medical diseases in low-income countries is 4.4 times higher than in high-income countries [45]. A vast majority of specialized emergency centers are usually localized in urban settings. In the United States, patients in rural settings are at significantly greater risk of death from traumatic and emergency cases when compared to their urban counterparts [46]. It then becomes an objective to bridge the gap between emergency care delivered to patients in rural settings and urban counterparts. A potential solution to bridge this gap lies in developing telemedicine for emergency and critical care. This would involve providing access for smaller and more rural healthcare facilities to specialists on an around-the-clock basis [47]. The implementation of telemedicine extends beyond video teleconferencing but also includes expert evaluation of patients and processes at remote locations. Several efforts have been made to expand on initial attempts to evaluate and treat patients needing remote emergency care. Mattox's group recorded the successful remote assessment of 17 trauma patients using real-time video observation coupled with verbal interaction [48]. Although this study was conducted in separate rooms within the same hospital, it showed that effective outcomes can be achieved even when the trauma team leader is not physically present during evaluation and initial resuscitation. Later research expanded this concept to remote settings. For example, a study on critical care in rural Vermont demonstrated how telemedicine consultations could help identify patient needs, guide diagnostic testing, and recommend therapeutic interventions [49]. Bidirectional video conferencing has been demonstrated in several settings of acute care to be an effective method for evaluation and teleconsultation [50,51]. For instance, the Southern Arizona Telemedicine and Telepresence program, launched in 2004, utilized secure real-time transmission of video, audio, and vital signs to connect a major university medical center with a rural facility located more than 100 miles away [52].

Telemedicine has also been extensively used for clinical consultations in real-time and specialties such as radiology, psychiatry, and dermatology to reduce the burden of care and extend specialty services to patients in underserved sites via teleradiology, telepsychiatry, and teledermatology, respectively [53-56]. In recent times, this application has shifted to more critical, life-threatening conditions like stroke, acute exacerbation of chronic obstructive pulmonary disease, and asthma [57,58]. To overcome the inequity of access to specialist stroke care, telemedicine systems have been devised to provide remote specialist assessment of patients with real-time clinical evaluation for hyperacute care and rehabilitation. For the past decade, telestroke has been a novel intervention for acute stroke treatment, in which the lack of access to stroke expertise has placed thrombolytic therapy out of reach for many patients [59,60]. Telestroke-assisted thrombolytic therapy compares favourably with face-to-face approaches, with no significant differences between survival and intracerebral hemorrhage in patients at risk for stroke [61]. The implementation of telestroke is evidence-based and recommended as a Class I intervention by the American Heart Association (AHA) [62]. It was also observed that telemedicine was effective in the early detection and proactive intervention in patients at home after an acute exacerbation of chronic obstructive pulmonary disease. It seems likely that adopting telehealth/telemedicine in everyday clinical practice could substantially improve the care of these patients, reducing morbidity, mortality, and health costs due to exacerbations [63]. Telemedicine consultation is also being used to coordinate and facilitate the management of patients requiring resuscitation. A study involving an aeromedical retrieval team in Australia demonstrated the significance of telemedicine in managing patients presenting with cardiac and respiratory arrests [64]. Meta-analyses have also suggested that telemonitoring, as a subset of telemedicine, in ambulatory patients with heart failure can improve mortality by 17-47% during 6-12 months of follow-up and reduce hospitalizations by 7-48% [65]. In accident and EDs, telemedicine has also been found to be a reliable method of assessing patients presenting with critical eye trauma [66].

Improving Efficiency and Workflow

One approach to addressing rising demand in emergency care is positioning a provider close to the triage area for immediate evaluation. ED crowding, room congestion, and unnecessary transfers can be averted through virtual triage. A descriptive analysis of a system in North Carolina found that the transfer rate for teleconsults was 32%, which was significantly higher than the background rate of transfers (5%) [67]. However, after adjusting for the severity of the injury, it was projected that 30% of the possible transfers were averted by telemedicine consultation. Screening becomes especially critical for patient safety during periods of high demand or in low-resource settings with limited staff, when patient volume and crowding hinder timely evaluation [68]. The application of telemedicine to screening poses an additional intervention. Through a real-time audio-visual interface between patients and remote care providers, telescreening optimizes providers' time, prioritizes care, and potentially minimizes expensive staffing requirements. Telemedicine has traditionally been used to connect minor treatment clinics to larger EDs and to facilitate specialty consultations [69]. Virtual communication and other telemedicine initiatives have been vital in streamlining communication between emergency providers and specialists. 

Enhancing Patient Outcomes

The potential of telemedicine to improve patient outcomes and access to emergency care is multifaceted. It solves many long-standing challenges such as geographical barriers, provider shortages, and the need for timely medical intervention [70]. Integrating telemedicine into emergency care has demonstrated the potential to reduce hospital admissions and improve triage efficiency [71]. Analysis of the impact of telemedicine on EMS suggests a reduction in mortality [72]. The use of telemedicine has also been shown to improve the process of transferring trauma patients from rural and remote locations [73]. Delays in transfer can lead to poor patient outcomes. Importantly, telemedicine can help to reduce the number of transfers by enabling trauma patients to be treated in rural hospitals [73]. This reduces unnecessary transports and admissions to an urban trauma center.

Challenges and barriers in telemedicine implementation

One of the key challenges in resource-constrained settings is the skewed distribution of healthcare delivery infrastructure [74]. While a large proportion of the population live in rural areas, healthcare establishments are located in urban settings. Some regions, especially in developing countries, still face limited access to stable internet networks and technological devices needed to run telemedicine services. This can be a major hindrance in bringing the benefits of telemedicine to remote areas and vulnerable communities. Another challenge in the implementation of telemedicine is the complex issue of regulation [75]. Each country has different regulations related to medical practices, patient privacy, and data security. The availability of sensitive health data is a significant issue in the implementation of telemedicine [76]. Ambiguous policies and differences in regulatory standards between countries can be a barrier to effectively implementing telemedicine on a global level. Acceptance from stakeholders, including medical personnel, patients, and healthcare institutions, is also a concern. Some medical personnel may feel uncomfortable with the use of technology in clinical practice, while some patients may doubt the safety and effectiveness of telemedicine services [77]. In addition, healthcare institutions also need to adapt their systems and procedures to integrate telemedicine into daily practice. From a legal perspective, factors such as state licensure, scope of practice, credentialing, professional liability, and patient confidentiality can all pose challenges to implementation. These issues must be carefully considered for the healthcare professional, the medical facility, and any potential service site [78]. Even within a single country, state boundaries can restrict the use of telemedicine in emergency care. For example, in Texas, USA, telemedical services are only permitted if the patient has had a prior in-person visit [79]. In Germany, only the state of North Rhine-Westphalia mandates that tele-emergency physicians complete a specialist course in tele-emergency medicine, which is accessible only after certain prerequisites are met.

Evidence supporting telemedicine in emergency care

Clinical Outcomes and Effectiveness

Telemedicine has demonstrated considerable promise in enhancing clinical outcomes across various emergency care domains. The telestroke model, for instance, has been pivotal in facilitating timely thrombolytic therapy for acute ischemic stroke patients in rural or underserved hospitals, significantly reducing door-to-needle times and improving functional outcomes [80,81]. Similarly, tele-ICU programs have been associated with reductions in mortality and length of stay, particularly in hospitals with limited intensivist coverage [82,83].

Beyond clinical metrics, telemedicine contributes to improved patient satisfaction by enhancing communication and reducing delays in care [84]. Safety outcomes also benefit; remote monitoring and real-time consultations ensure continuity of care and reduce the risk of medical errors during interfacility transfers or in settings with limited specialist access [85]. Notably, patients and families often express high levels of satisfaction with virtual consultations, citing convenience and timeliness as key advantages [86].

Operational Efficiency

From an operational standpoint, telemedicine addresses long-standing challenges such as ED overcrowding and staff shortages. By enabling offsite clinicians to support triage and initial assessment, telemedicine encounters enhance the operational flow of emergency care while avoiding increased rates of death, hospitalization, or frequent ED visits [87].

Telemedicine also optimizes resource utilization. Real-time access to remote experts enables faster decision-making, limits unnecessary imaging or admissions, and reduces staff burden in high-volume settings [88]. Integration with electronic health records and decision-support systems further enhances the efficiency and safety of care delivery [89].

Global Success Stories

Around the world, telemedicine has enabled innovative models of emergency care delivery. In India, the "eSanjeevani" initiative has expanded access to emergency consultations in rural areas, with over 200 million consultations recorded to date [90]. In the United States, health systems have successfully embedded tele-ICU services in regional hospitals, achieving significant quality improvements [91]. Likewise, sub-Saharan African countries such as Rwanda have piloted mobile health (mHealth) and emergency teleconsultation services with notable success, despite infrastructural limitations [92].

Differences in implementation between high- and low-resource settings underscore the importance of contextual adaptation. While developed regions benefit from robust digital infrastructure, developing countries often rely on mobile platforms and community health worker integration to extend emergency services [93]. Nonetheless, success stories across diverse contexts reaffirm telemedicine's adaptability and utility in emergency care globally.

Innovations and future directions

Emerging Technologies

The future of telemedicine in emergency care is deeply intertwined with the advancement of emerging technologies. Artificial intelligence (AI) and machine learning are increasingly integrated into tele-triage systems, enabling rapid risk stratification and clinical decision support [94]. For instance, AI-driven platforms can analyze vital signs, symptoms, and medical history to prioritize high-risk patients for immediate intervention.

Wearable devices and remote monitoring tools are revolutionizing patient engagement and post-discharge follow-up. These tools allow clinicians to track physiological parameters in real time, enabling the early detection of deterioration in patients with chronic or acute conditions [95]. Such technologies not only improve outcomes but also reduce unnecessary hospital readmissions [96].

Improving Accessibility and Scalability

One of the most critical challenges for telemedicine is ensuring equitable access. Innovations aimed at low-resource settings include solar-powered telemedicine kits, mobile teleconsultation vans, and community-based hubs with minimal digital infrastructure requirements [97]. Open-source platforms and low-bandwidth technologies also hold promise for expanding access in underserved areas.

Bridging the digital divide requires investment in digital literacy, affordable internet, and device accessibility. Collaborative efforts, involving governments, nongovernmental organizations (NGOs), and the private sector, have shown success in scaling up telemedicine programs in resource-constrained environments [98].

Policy Evolution and Standardization

As telemedicine becomes more entrenched in clinical practice, regulatory frameworks must evolve to provide clarity and consistency. Cross-state licensure in the United States, data privacy regulations such as the General Data Protection Regulation (GDPR), and standardized protocols for remote care are key considerations [99]. Policy harmonization is particularly important in emergency care, where timely intervention is critical and delays due to legal ambiguities can be detrimental.

Equally important is the development of reimbursement models that recognize the value of virtual care. Policies that incentivize telemedicine adoption through insurance coverage, bundled payments, or grants can accelerate integration into mainstream emergency practice [100].

Future Research Priorities

Despite its growth, telemedicine remains underexplored in certain dimensions. Long-term outcomes, particularly in relation to chronic disease management after emergency interventions, require more robust investigation [101]. Comparative effectiveness studies are essential to determine the contexts in which telemedicine provides the most value.

In light of recent global health crises, there is also growing interest in telemedicine's role in disaster and pandemic response. From remote triage during COVID-19 surges to mental health support during humanitarian emergencies, telemedicine has proven indispensable, yet more evidence is needed to formalize these strategies into emergency preparedness plans.

## Conclusions

Telemedicine has emerged as a transformative force in emergency and critical care, offering measurable improvements in clinical outcomes, patient satisfaction, and operational efficiency. From tele-stroke networks to remote ICU monitoring, its impact spans various domains and geographic regions, bridging gaps in access and expertise. Innovations such as AI-driven diagnostics, wearable health monitors, and mobile platforms continue to push the boundaries of what is possible, while ongoing efforts to ensure scalability and accessibility promise broader inclusion. As regulatory frameworks evolve, standardization and sustainable reimbursement models will be key to embedding telemedicine into everyday practice. Moreover, future research must address existing knowledge gaps, particularly in long-term outcomes and disaster response. To fully harness the potential of telemedicine, a concerted, collaborative effort is needed, one that brings together clinicians, policymakers, technologists, and communities. In doing so, telemedicine can truly become a cornerstone of resilient, responsive, and equitable emergency care systems worldwide.
